# Supplementary material for: Mutationally-activated PI3’-kinase-α promotes de-differentiation of lung tumors initiated by the BRAFV600E oncoprotein kinase
Source: eLife. 2019 Aug 27;8:e43668. doi: 10.7554/eLife.43668 (PMC6711745; doi:10.7554/eLife.43668)
Supplement: Transparent reporting form [file elife-43668-transrepform.docx]

***eLife’s* transparent reporting form**

We encourage authors to provide detailed information *within their submission* to facilitate the interpretation and replication of experiments. Authors can upload supporting documentation to indicate the use of appropriate reporting guidelines for health-related research (see [EQUATOR Network](http://www.equator-network.org/%20)), life science research (see the [BioSharing Information Resource](https://biosharing.org/)), or the [ARRIVE guidelines](http://www.plosbiology.org/article/info:doi/10.1371/journal.pbio.1000412) for reporting work involving animal research. Where applicable, authors should refer to any relevant reporting standards documents in this form.

If you have any questions, please consult our Journal Policies and/or contact us: [editorial@elifesciences.org](mailto:editorial@elifesciences.org).

**Sample-size estimation**

- You should state whether an appropriate sample size was computed when the study was being designed
- You should state the statistical method of sample size computation and any required assumptions
- If no explicit power analysis was used, you should describe how you decided what sample (replicate) size (number) to use

Please outline where this information can be found within the submission (e.g., sections or figure legends), or explain why this information doesn’t apply to your submission:

Power analysis was done for mouse experiments as outlined in methods under “Experimental model: Mice.” For in vitro experiments, necessary replicates were modeled on previously published work.

**Replicates**

- You should report how often each experiment was performed
- You should include a definition of biological versus technical replication
- The data obtained should be provided and sufficient information should be provided to indicate the number of independent biological and/or technical replicates
- If you encountered any outliers, you should describe how these were handled
- Criteria for exclusion/inclusion of data should be clearly stated
- High-throughput sequence data should be uploaded before submission, with a private link for reviewers provided (these are available from both GEO and ArrayExpress)

Please outline where this information can be found within the submission (e.g., sections or figure legends), or explain why this information doesn’t apply to your submission:

A definition of biological vs technical replication is provided at the beginning of the methods section. For imaging experiments, replication numbers are listed in methods under “Microscopy and Quantitation of Immunofluorescent Images**.**” For sequencing and GSEA analyses, replicates are listed in methods under “Experimental Model: Mice.” For luciferase assays in figure 7, replicates are listed in methods under “Luciferase Assays.” For western blots and co-immuno precipitation experiments in figure 7, replicates are listed in methods under “Protein Biochemistry.” For bioinformatic analysis of human lung adenocarcinoma in figure 5 – supplement 1, replicates are listed in methods under “Bioinformatics.” No samples or animals were excluded as “outliers” because of experimental results.

**Statistical reporting**

- Statistical analysis methods should be described and justified
- Raw data should be presented in figures whenever informative to do so (typically when N per group is less than 10)
- For each experiment, you should identify the statistical tests used, exact values of N, definitions of center, methods of multiple test correction, and dispersion and precision measures (e.g., mean, median, SD, SEM, confidence intervals; and, for the major substantive results, a measure of effect size (e.g., Pearson's r, Cohen's d)
- Report exact p-values wherever possible alongside the summary statistics and 95% confidence intervals. These should be reported for all key questions and not only when the p-value is less than 0.05.

Please outline where this information can be found within the submission (e.g., sections or figure legends), or explain why this information doesn’t apply to your submission:

All P-values listed in figures, with statistical details in figure legends. Exact P values listed for all key tests except when P < .0001. N values listed in methods as described above.

(For large datasets, or papers with a very large number of statistical tests, you may upload a single table file with tests, Ns, etc., with reference to sections in the manuscript.)

**Group allocation**

- Indicate how samples were allocated into experimental groups (in the case of clinical studies, please specify allocation to treatment method); if randomization was used, please also state if restricted randomization was applied
- Indicate if masking was used during group allocation, data collection and/or data analysis

Please outline where this information can be found within the submission (e.g., sections or figure legends), or explain why this information doesn’t apply to your submission:

Animals were randomized into groups prior to tumor induction as described in “Experimental Model: Mice.”

**Additional data files (“source data”)**

- We encourage you to upload relevant additional data files, such as numerical data that are represented as a graph in a figure, or as a summary table
- Where provided, these should be in the most useful format, and they can be uploaded as “Source data” files linked to a main figure or table
- Include model definition files including the full list of parameters used
- Include code used for data analysis (e.g., R, MatLab)
- Avoid stating that data files are “available upon request”

Please indicate the figures or tables for which source data files have been provided:

R script and source data has been included for GSEA analyses shown in figures 2,3, and 7. R script and source data has been included for WGCNA clustering shown in figure 6. R script and source data have been included for quantitated immunofluorescence images. Numerical data and statistical testing has been included for luciferase assays in figure 6. Cellprofiler pipelines have been included for quantifications of immunostaining shown in figures 3,4,5,7, and Figure 4 – figure supplement 1. All source data provided with manuscript, all R scripts available at github.com/jevanveen/vanveen-elife
